# Host–Gut Microbiota Interactions: Exploring the Potential Role of Vitamin B1 and B2 in the Microbiota–Gut–Brain Axis and Anxiety, Stress, and Sleep Quality

**DOI:** 10.3390/nu17111894

**Published:** 2025-05-31

**Authors:** Yingxuan Tao, Murong Wu, Boyao Su, Heng Lin, Qianzi Li, Yuhong He, Tian Zhong, Ying Xiao, Xi Yu

**Affiliations:** 1 Faculty of Medicine, Macau University of Science and Technology, Avenida Wai Long Taipa, Macau; yingxuan.tao@outlook.com (Y.T.); 2240002221@student.must.edu.mo (M.W.); 2230017878@student.must.edu.mo (B.S.); 2230004854@student.must.edu.mo (H.L.); 2230027731@student.must.edu.mo (Q.L.); tzhong@must.edu.mo (T.Z.); yxiao@must.edu.mo (Y.X.); 2 Faculty of Chinese Medicine, Macau University of Science and Technology, Avenida Wai Long Taipa, Macau; heyuhong430@gmail.com

**Keywords:** gut microbes, microbiota–gut–brain axis, host–microbiota interaction, vitamin B1 and B2, brain-related mental health

## Abstract

*Background:* The microbiota–gut–brain axis plays a key role in regulating mental health, including anxiety, stress, and sleep quality. Vitamins B1 and B2 may influence these outcomes by modulating gut microbiota. This study aimed to examine the relationships among mental health indicators, gut microbiota, and levels of vitamins B1 and B2. *Methods*: This study conducted a cross-sectional analysis to explore associations between mental health status, gut microbiota composition and function, and circulating vitamin B1/B2 levels. Ten representative microbes were selected for analysis. Mediation models were used to assess whether gut microbiota mediate the effects of vitamins on mental health. *Results:* Vitamin B1 and B2 levels were significantly associated with stress, sleep quality, and sleepiness (*p* < 0.05). The abundance of specific gut microbiota also showed significant inter-correlations (*p* < 0.05). Specific gut microbiota abundances are correlated with host anxiety, stress, sleep, and sleepiness levels (*p* < 0.05). We did not observe significant differences in the abundance of specific gut microbiota associated with different vitamin B1 and B2 nutritional levels in the host (*p* > 0.05). Gut microbial diversity and composition varied notably between different vitamin level groups and anxiety, stress, sleep quality, and sleepiness groups. Although both vitamin B2 and *Bacteroides* had significant direct effects on sleep quality (*p* < 0.05), no mediating effect of *Bacteroides* was observed (*p* > 0.05). *Conclusions:* These findings suggest potential associations between vitamins B1 and B2 and mental health, as well as between gut microbiota and host psychological outcomes; no significant mediating effect of the microbiota was observed. These exploratory results offer preliminary insights for future research on microbiota-targeted interventions and precision nutrition strategies.

## 1. Introduction

Anxiety is the most prevalent mental illness [1]. A global systematic assessment covering 204 countries and territories in the context of the COVID-19 pandemic in 2020 estimated an additional 76.2 million (64.3 to 90.6 million) cases of anxiety disorders worldwide because of the pandemic [2]. One of the most prevalent health issues facing people today is stress, which has been dubbed the “disease of the century” [3]. High levels of stress are linked to a higher risk of anxiety, even though stress is not a mental illness in and of itself [4]. Individual survival and growth depend on sleep, which is the most basic living process in biological evolution [5]. A meta-analysis reported a 24.1% prevalence of sleep disorders among Chinese university students [6]. Another meta-analysis estimated the prevalence of sleep disorders in the general Chinese population at 19%, making poor sleep quality and sleep disorders a significant public health concern [7]. As a result, anxiety, stress, and sleep disorders need proper attention because they not only have an impact on health and quality of life, but they also place a significant burden on families and society.

Uncertainty surrounds how anxiety, stress, and sleep disturbances work. On the other hand, neurotransmitters and oxidative stress may be linked to stress and anxiety. Numerous neurotransmitters, including norepinephrine, gamma-aminobutyric acid (GABA), serotonin, and stress and anxiety, have been proposed by some researchers to interact with one another. As the main inhibitory neurotransmitter, GABA helps people feel less stressed and anxious. On the other hand, long-term stress and anxiety can throw off neurotransmitter homeostasis [8]. According to other researchers, excessive stress and anxiety cause the body’s redox regulation to be disrupted, which encourages the release of stress hormones like cortisol and epinephrine. This causes dysfunctional mitochondria to produce too many reactive oxygen species (ROS), which weakens the body’s antioxidant defenses [9]. The overproduction of ROS harms proteins, DNA, and cell membranes and speeds up the start of lipid peroxidation and membranes of cells. Malondialdehyde, 4-hydroxynonenal (4-HNE), and other lipid peroxidation products might cause inflammation [10]. This exacerbates oxidative stress-induced brain damage by generating a vicious loop of ROS, RNS, and mitochondrial dysfunction [9]. Furthermore, according to some experts, the development of stress and anxiety may be linked to micronutrient deficits [11]. Stress and anxiety significantly impair the quality of sleep. Stress and anxiety make the brain persistently hyperactive, which makes it hard to fall asleep. Stress causes the body to produce stress hormones and trigger a stress response, which impair brain function and lower the proportion of deep sleep, resulting in sleep deprivation [12]. Furthermore, sleep disturbances may raise the risk of metabolic illnesses, cardiovascular diseases, weakened immune systems, and problems of the intestinal flora, in addition to psychiatric conditions including anxiety and depression [13].

The present treatments for reducing stress and anxiety and enhancing sleep quality are not totally successful, in addition to having negative side effects and being expensive. Because of their safe and natural qualities, alternative medicines like vitamins, minerals, and nutritional supplements have become more popular [14]. Vitamins B1 and B2 are both necessary micronutrients. Through their ability to fight oxidative stress, enhance neurotransmitter synthesis, and promote cellular energy metabolism, vitamins B1 (thiamine) and B2 (riboflavin) protect brain areas linked to mood and sleep disorders [3]. This implies that mood swings and sleepiness may be related to vitamin B1 and vitamin B2. Thiamine and riboflavin supplements can help reduce stress and anxiety while enhancing the quality of sleep. Adjunctive supplementation with thiamine and vitamin B6 was linked to improved sleep quality, reduced anxiety, and cognitive impairment, according to an intervention trial [15]. A double-blind, randomized controlled experiment with a placebo has shown that thiamine nutritional supplementation by itself was successful in reducing anxiety and depression in patients with major depression [16]. A clinical study has shown that night shift workers’ sleep quality was enhanced by taking supplements containing a mix of nutrients and meals high in vitamin B [17]. However, a cross-sectional study by Baharak et al. found that a reduced prevalence of stress and anxiety was linked to the dietary intake of vitamin B in the study population [18]. The potent antioxidant properties of riboflavin have been the subject of numerous clinical studies. According to a multicenter randomized clinical research project, succinic acid and riboflavin demonstrated significant promise in reducing stress and anxiety by boosting the activity of antioxidant enzymes to lessen diabetic peripheral neuropathy (DPN) issues [19]. As far as we are aware, vitamin supplementation has always been a popular area of study, although the majority of these studies have concentrated on certain vitamin groups or well-known vitamins (e.g., vitamin C, vitamin D, vitamin B6, etc.). Research on vitamins B1 and B2 has received less attention. Fewer research projects have examined thiamine specifically; the majority of thiamine studies have simply assessed the B vitamin group in general. The majority of riboflavin research investigations have been on the extent to which riboflavin can be employed in clinical trials to relieve nerve discomfort and diabetic nephropathy (DN). We therefore set out to investigate the effects of concurrent thiamine and riboflavin supplementation on reducing anxiety and stressful conditions and enhancing the quality of sleep in young individuals, considering the limits and inconsistencies of data from earlier studies.

The emerging roles of thiamine and riboflavin in gut microbial ecology and intestinal homeostasis are increasingly being recognized [20]. The human gut microbiota is an independent, relatively stable, and dynamic ecosystem, primarily composed of the *Bacteroidetes* phylum, *Firmicutes* phylum, *Actinobacteria* phylum, *Proteobacteria* phylum, and a small proportion of *Verrucomicrobia* phylum [21]. Many gut bacterial strains could synthesize free thiamine or TPP, and over 90% of *Bacteroidetes* carry genes related to thiamine synthesis or transport [22]. Research suggests that thiamine regulation of the TCA cycle may also influence gut-associated immune cell function [23]. Genomic analyses indicate all the *Bacteroidetes*, *Fusobacteria*, and 36 genomes (92%) of *Proteobacteria* phyla contain all the basic functional roles of riboflavin biosynthesis [24]. While approximately half of *Firmicutes* are predicted to be riboflavin producers, some species lack the RibU transporter and thus rely on exogenous sources [24]. Previous studies suggest that both thiamine and riboflavin are closely linked to gut microbial composition and function, which are critical for maintaining intestinal health.

Numerous studies on the microbiota of humans and animals in recent decades have demonstrated the critical role gut microbiota play in fostering health and controlling the growth and operation of the central nervous system [25]. Human brain function and health are influenced by a complex system of bidirectional signals between the gut microbiota and the brain. To produce pro- or anti-inflammatory responses, such as the release of pro-inflammatory cytokines (*IL-6*, *TNF-alpha*), immune cell populations in the gastrointestinal tract are continuously interacting with microbes and their metabolites. These interactions can result in gut–brain signaling. These interactions enter the bloodstream and lymphatic circulations and impact the entire body, including the brain [26]. The brain nerve that allows for two-way communication between the gut and the brain is called the vagus nerve [27]. By controlling the gut microbiota, the vagus nerve affects neurodevelopment, emotional and social issues associated with mood disorders, and serotonin and dopamine neurotransmission in the brain [28,29]. Furthermore, through chemicals that the gut microbiota modulates, it can affect host–microbiota interaction. Numerous investigations have demonstrated the critical role that short-chain fatty acids—such as propionate, butyrate, and acetate—play in gut–brain communication pathways [30]. Overall, the microbiota–gut–brain axis affects the host’s cerebral nerves, anxiety, stressful emotions, sleep quality, and cognitive impairment through interactions with neuronal, endocrine, immunological, and metabolic pathways.

Therefore, this study aims to explore the relationship between nutritional levels of vitamin B1 and B2 and psychological outcomes including anxiety, stress, and sleep quality, based on their established antioxidant and neuroprotective properties. Additionally, given the crucial role of the gut microbes in modulating brain function through the microbiota–gut–brain axis, we further examined how gut microbial composition is associated with these psychological variables. A pathway analysis model was constructed to investigate whether specific gut microbes mediate the effects of vitamin B1 and B2 on anxiety, stress, and sleep quality.

## 2. Materials and Methods

### 2.1. Experimental Design

This study utilized a cross-sectional research design with the aim of exploring the relationship between vitamin B1 and vitamin B2 nutritional levels, gut microbiota, and anxiety, stress, and sleep quality. This study was conducted from March 2024 to February 2025 at Macau University of Science and Technology (MUST), Macau, and included 76 participants.

### 2.2. Participants

Researchers recruited male and female students aged 18–25 years from Macau University of Science and Technology, China, through campus advertisements and social media. Interested participants were first screened through on-site screening and were required to meet the following inclusion criteria: (1) age 18–25 years; (2) no chronic digestive disease; (3) not taking probiotic or vitamin supplements for at least 3 months; and (4) able to provide a urine or fecal sample and complete an assessment scale. The exclusion criteria were as follows: individuals with (1) a BMI below 18.5 and over 40 kg/m^2^; (2) individuals with a long history of medication (antibiotics, antidepressants, etc.); (3) individuals who had recently experienced a major life event (e.g., surgery, bankruptcy, failure in love, etc.), as well as a history of the death of a first-degree relative and close friend in the past six months; (4) individuals with neurological or organic disorders; (5) individuals with pathologic anxiety (e.g., generalized anxiety disorder GAD) or psychiatric disorders (e.g., post-traumatic stress disorder); (6) individuals with a diagnosis of an inflammatory disorder and other specified disorders such as diabetes mellitus, cardiovascular, cancer, hypertension, renal disease, liver disease, hyperthyroidism, epilepsy, and multiple sclerosis; (7) individuals with a diagnosis of a pathologically based sleep disorder (e.g., severe insomnia, severe obstructive breathing sleep apnea syndrome); (8) individuals with a recent history of alcohol and drug use (past 3 months); (9) pregnant or breastfeeding females; and (10) individuals with allergies to VBI and VB2 tablets. In addition, subjects who failed to complete the questionnaire and follow the guidelines correctly were excluded.

To find participants who met the criteria, 105 participants who met the inclusion criteria were first introduced to the study, and 5 participants withdrew (1 refused to participate and 4 could not be contacted). Then, 100 participants were invited to complete the Self-Assessment Scale for Anxiety (SAS) and the Perceived Stress Scale (PSS) to assess anxiety and stress status and the Pittsburgh Sleep Quality Index (PSQI) and the Epworth Sleepiness Scale (ESS) to assess sleep quality. All urine samples from the 4 h vitamin urinary loading test were collected and subjected to laboratory assays for nutritional levels of vitamins B1 and B2. Their fecal samples were also collected. Twenty-four participants dropped out (four did not submit the scale at the designated time, six did not submit the 4 h urine sample, and fourteen had highly repetitive responses that were determined to constitute invalid assessments). Finally, 76 participants met the inclusion and exclusion criteria to be selected as participants and included in the vitamin series study, of which 54 submitted qualifying fecal samples to be included in the gut microbiota series study (of the 76 individuals, 10 did not submit fecal samples at the specified time and 12 submitted non-qualifying fecal samples).

### 2.3. Sample Collection and Processing

(1) Vitamin B1 and B2 4 h urinary load test and assays

A total of 76 participants were instructed to empty their bladders of urine in the morning, then take 1 tablet of vitamin B1 (5 mg) and 1 tablet of vitamin B2 (5 mg) orally, and drink an adequate amount of warm water (200–400 mL) during the waiting period while using a urine bag to collect urine. Participants were instructed to collect all urine produced within 4 h of taking the medication (including the last urine sample upon arrival). After collecting all urine, we measured the urine volume. Researchers acidified the urine to maintain a pH of 4–5 to ensure vitamin stability. The acidified urine samples were immediately transported to the laboratory for testing, labeling, and storage at 4 °C [31]. Vitamin B1 and vitamin B2 in urine were measured in the laboratory of the Faculty of Medicine of the Macau University of Science and Technology. Due to the fluorescent properties of vitamin B1 and vitamin B2, their fluorescent signals can be detected by an enzyme labeling instrument (MEGAMAN^®^ (Shanghai, China) Co., Ltd., SpectraMax iD3 Multi-Mode). Vitamin B1 can be measured at the excitation wavelength of 365 nm and emission wavelength of 435 nm; vitamin B2 can be measured at the excitation wavelength of 420 nm and emission wavelength of 530 nm for the fluorescence signal. The amount of vitamin B1 or vitamin B2 in a urine sample is calculated from a pre-drawn standard curve. A urinary vitamin B1 level of <100 μg is considered lacking, 100–200 μg is insufficient, and >200 μg is normal [32,33]; a urinary vitamin B2 level of <400 μg is considered lacking, 400–799 μg is insufficient, 800–1300 μg is normal, and >1300 μg is considered adequate [31,32].

(2) fecal sample collection and quantitative analysis of specific gut microorganisms

The researcher trained the participants to collect fecal samples beforehand to ensure that interfering factors such as urine and food residues were reduced during the collection process. Participants collected their own fecal samples using a sterile sampling spoon and collection tube. After sampling, samples were transported to the laboratory by researchers within 6 h, numbered, registered, and stored at −80 °C for cryogenic freezing. Microbial DNA was extracted from the feces of 54 volunteers using the Aidlab Fecal Genomic DNA Extraction Kit (Aidlab Biotechnologies Co., Ltd., Beijing, China), as described in Supplementary Material A. The concentration of total fecal microbial DNA (Ct) in each sample DNA eluate were detected using Nanodrop One (Thermo Fisher Scientific Inc., Waltham, MA, USA). According to the study by Wu et al., we used real-time qPCR to quantify ten specific gut microbes in the gut. These 10 representative microbes are considered potential indicators for assessing the entire human gut microbiome, including probiotics (*Lactobacillus* and *Bifidobacterium*), opportunistic pathogens (*Enterobacteriaceae*, *Enterococcus*, *Bacteroides*, and *Atopobium*), and other health-promoting symbiotic bacteria (*Faecalibacterium prausnitzii* (*F. prausnitzii*), *Clostridium butyricum* (*C. butyricum*), *Clostridium leptum* (*C. leptum*), and *Eubacterium rectale* (*E. rectale*) [34]. Primers were synthesized by Sangon Biologicals (Sangon Biotech Co., Ltd., Shanghai, China), and the primer information is shown in Supplementary Materia B Table S1. qPCR was performed on 54 fecal bacterial DNA samples with a reaction volume of 10 μL using the Step One Plus Real Time PCR System (Applied Biosystems, Inc., Foster City, CA, USA), which included 5 μL of SYBR Green PCR High Rox premix (Aidlab, China), 0.2 μL of primer pairs (0.2–0.6 μmol-L-1), 3.6 μL of Depc water, and 1 μL of template DNA or 1 μL of distilled water (negative control). The reaction conditions are listed in Supplementary Materia B Table S2. Each reaction was performed in triplicate, and the cycling threshold (ΔCT) between replicates was required to be less than 0.5. Plasmid DNA standards containing the corresponding amplified fragments for each primer set were diluted in a 10-fold gradient and amplified with the bacterial DNA template in the same PCR plate. The copy number of the target bacteria in the DNA template is determined by comparing the standard curve obtained by amplification with the corresponding bacterial DNA standard. The final concentration of target bacteria is obtained by dividing the concentration of target bacteria in the DNA template (N) by the total DNA concentration of fecal microorganisms in the DNA eluate of each sample and the volume of template (V). Concentrations are expressed in copies per nanogram of total DNA of fecal microorganisms, hereafter referred to as copy number-ng-1. The calculation formula is listed in the Supplementary Materia C.

### 2.4. Scale

(1) Self-Rating Anxiety Scale (SAS)

The Self-Rating Anxiety Scale (SAS), developed by American psychologist Zung in 1971, is a self-report scale used to assess an individual’s feelings of anxiety [35]. The SAS scale consists of a total of 20 items, which assess an individual’s symptoms of anxiety over the past week. The items in the scale cover somatic and emotional responses to anxiety, including nervousness, fear, rapid heartbeat, difficulty sleeping, and lethargy. Each entry consists of ratings of 1 (no or very little time), 2 (a small amount of time), 3 (quite a bit of time), and 4 (most or all of the time). Participants answered questions based on their feelings of anxiety. A raw total score was first calculated for each scale, and the raw score was multiplied by 1.25 to obtain a standardized score, which was a number between 25 and 100.

(2) Perceived Stress Scale (PSS)

Developed by Cohen et al. in 1983, the Perceived Stress Scale is designed to measure an individual’s subjective experience and perception of life stress over the past month and is a psychometric tool used to assess an individual’s perceived level of stress, which is widely used in clinical assessment and mental health research [36]. The items in the scale cover an individual’s perception of stress in different situations in daily life, including the sense of control over life events, the frequency of stress, and the ability to cope with life events. Each item includes ratings of 0 (never), 1 (rarely), 2 (sometimes), 3 (often), and 4 (always). Participants answered these questions based on their own sense of stress, and a total score, a number between 0 and 40, was calculated for each scale.

(3) Pittsburgh Sleep Quality Index (PSQI)

The Pittsburgh Sleep Quality Index was developed in 1989 by Buysse et al. at the University of Pittsburgh, USA, as a self-report scale for assessing the quality of an individual’s sleep [37]. The PSQI has 19 entries with 7 components, including subjective sleep quality, sleep latency, sleep duration, sleep efficiency, sleep disorders, use of sleep medication, and daytime dysfunction. The rating range for each component included 0 (none), 1 (<1 time/week), 2 (1–2 times/week), and 3 (≥3 times/week). Participants answered these questions based on their sleep reality, and a scale score was calculated as a number between 0 and 21. Higher scores indicate poorer sleep quality.

(4) Epworth Sleepiness Scale (ESS)

Developed by Johns in Australia in 1991, the Epworth Sleepiness Scale is a self-report scale used to measure an individual’s daytime sleepiness [38]. Designed to help identify possible sleep disorders, it can be used as a supplement to the daytime dysfunction component of the PSQI. The ESS consists of eight scenarios, each of which represents a daytime scenario that could lead to drowsiness. Each scenario is rated on a scale ranging from 0 (will not doze off), 1 (little likelihood of dozing off), 2 (moderate likelihood of dozing off), and 3 (very likely to doze off). Participants answered these questions based on their actual daytime sleepiness, and a scale score was calculated.

### 2.5. Statistical Analysis

R software (version 4.3.2; R Core Team, 2023) was used to process and plot all statistical analyses and visualizations in this study, primarily utilizing the R extension packages ggplot2 (version 3.5.1) and corrplot (version 0.95). First, the distribution of the observations was examined, and the normality of the data was tested. To compare the differences in vitamin, anxiety, stress, sleep, and sleepiness between male and female groups, an independent samples *t*-test was used. For continuous variables that did not meet normal distribution, the Mann–Whitney U test was applied. A one-way ANOVA and Kruskal–Wallis H test was used to compare differences in anxiety, stress, sleep, and sleepiness scores across different vitamin B1 and B2 levels. Additionally, to further assess the correlation between specific gut microbiota abundance and host’s anxiety, stress, sleep quality, and sleepiness status, Spearman’s correlation analysis was performed. To compare the differences in gut microbiota among the different anxiety, stress, sleep, and sleepiness groups, we performed a one-way ANOVA and Kruskal–Wallis H test. Based on the results of the one-way ANOVA, we used the LSD test to further compare pairwise differences between the groups. We conducted a one-way ANOVA and Kruskal–Wallis H test to compare the differences in gut microbiota among the different Vitamin B1 and B2 level groups. Finally, to explore whether the effects of vitamins on mental health were mediated by gut microbiota, a mediation analysis was conducted within a path analysis framework using structural equation modeling (SEM) syntax. All statistical analyses were performed using *p* < 0.05 as the criterion for statistical significance.

### 2.6. Ethical Considerations

This study was approved by the Medical Ethics Committee of the Macau University of Science and Technology, Macau, under the ethical guideline MUST-FDCT-20240226001. Each participant was enrolled freely with informed consent and was free to quit at any time during this study without incurring any fees, in accordance with the principle of voluntary participation. Informed consent was signed by each subject. Human sample collection, processing, and preservation—including urine and feces—were performed strictly in accordance with ethical standards, laboratory rules, and the relevant Declaration of Helsinki guidance. With the thorough documentation of sample collection, processing, storage, and use, the research team has put in place a methodical sample management system. When samples are no longer needed, they are disposed of in compliance with the laboratory’s defined destruction protocols to guarantee a secure, uniform, and moral disposal.

## 3. Results

### 3.1. Baseline Characteristics of Participants

Table 1 describes the baseline characteristics of the study population (n = 76). The mean ± SD age of the participants was 19.5 ± 1.2 years, with 52.6% males and 47.4% females. Approximately 84.2% of participants had light daily physical activity, 90.7% were nonsmokers, and 92.1% did not drink alcohol. The mean ± SD BMI of the participants was 21.3 ± 3.1. The mean ± SD urinary level of vitamin B1 was 269.2 ± 382.8 µg. The mean ± SD urinary level of vitamin B2 was 321.3 ± 365.5 µg. The participants had a mean ± SD anxiety score of 42.5 ± 9.9, mean ± SD stress score of 19.6 ± 4.9, mean ± SD sleep quality index of 9.2 ± 4.8, and mean ± SD sleepiness score of 10.2 ± 5.7.

### 3.2. Effects of Vitamin B1 and B2 Levels on Anxiety, Stress, and Sleep Quality

(1) Statistically significant differences in anxiety and stress levels between male and female groups

The independent samples *t*-test was used to analyze the differences between males and females in vitamin B2 levels, anxiety (SAS scale), perceived stress (PSS scale), sleep quality (PSQI scale), and sleepiness (ESS scale). The results showed that there were no statistically significant differences in vitamin B2 levels (t = −0.36, 95% CI: −90.77–63.29, *p* = 0.723), PSQI scores (t = −0.42, 95% CI: −2.72–1.76, *p* = 0.674), and ESS scores (t = −0.48, 95% CI: −3.23–1.97, *p* = 0.631), and there was no statistically significant difference between men and women. However, women had significantly higher SAS scores (t = −3.78, 95% CI: −12.36–(−3.79), *p* < 0.05) and PSS scores (t = −3.33, 95% CI: −5.62–(−1.43), *p* < 0.05), and there was a statistically significant difference in the levels of anxiety and stress between the male and female groups (Table 2).

The Mann–Whitney U test was used to compare vitamin B1 levels between the male and female groups. The results showed that the mean rank (mean rank) was 40.00 for males and 36.83 for females, and the difference between the two groups was not statistically different (U = 660.00, Z = −0.624, *p* = 0.533).

(2) Statistically significant differences in sleep quality and drowsiness scores by vitamin B1 level

The variations in anxiety (measured by the SAS scale), stress (measured by the PSS scale), sleep quality (measured by the PSQI scale), and sleepiness (measured by the ESS scale) among the groups with varying vitamin B1 levels were investigated using the Kruskal–Wallis H test. The results showed that SAS scores (H = 0.541, *p* = 0.978) and PSS scores (H = 8.469, *p* = 0.075) were not statistically different between groups with different vitamin B1 levels (*p* > 0.05). However, the PSQI score (H = 34.087, *p* < 0.05) and ESS score (H = 37.667, *p* < 0.05) were statistically different between groups with different vitamin B1 levels (Table 3).

(3) Statistically significant differences in drowsiness scores between different vitamin B2 levels

A one-way ANOVA was used to test the differences in anxiety (SAS scale), stress (PSS scale), sleep quality (PSQI scale), and sleepiness (ESS scale) in different vitamin B2 level groups. The results showed that SAS scores (F = 0.215, *p* = 0.886), PSS scores (F = 1.190, *p* = 0.320), and PSQI scores (F = 2.641, *p* = 0.056) were not statistically different between the different vitamin B2 level groups (*p* > 0.05). However, there was a statistically significant difference in ESS score (F = 3.561, *p* = 0.018) between groups with different vitamin B2 levels (Table 4).

(4) Vitamin B1 levels were associated with stress, sleep quality, and drowsiness; vitamin B2 levels were associated with sleepiness

Spearman’s correlation analysis was used to explore the correlation between vitamin B1 and vitamin B2 on the scores of SAS, PSS, PSQI, and ESS scales. As shown in the table, vitamin B1 was positively correlated with the PSS scale (ρ = 0.341, *p* = 0.003) and negatively correlated with the PSQI scale (ρ = −0.592, *p* < 0.05) and the ESS scale (ρ = −0.654, *p* < 0.05). In contrast, the correlation between vitamin B1 and the SAS scale (ρ = 0.002, *p* = 0.985) was not significant. Vitamin B2 was negatively correlated with the ESS scale (ρ = −0.235, *p* = 0.041), whereas the correlations with the SAS scale (ρ = −0.001, *p* = 0.993), the PSS scale (ρ = 0.149, *p* = 0.199), and the PSQI scale (ρ = −0.223, *p* = 0.053) were not significant. In addition, we found a positive correlation between the SAS scale and the PSS scale (ρ = 0.357, *p* = 0.002), and a positive correlation between the PSQI scale and the ESS scale (ρ = 0.847, *p* < 0.05). A positive correlation was also shown between vitamin B1 and vitamin B2 levels (ρ = 0.282, *p* = 0.014) (Table 5).

### 3.3. Association of Specific Gut Microbes with Anxiety, Stress, and Sleep Quality: Heat Map and Network Analysis

(1) Distribution of specific gut microbial abundance

**Figure 1:**
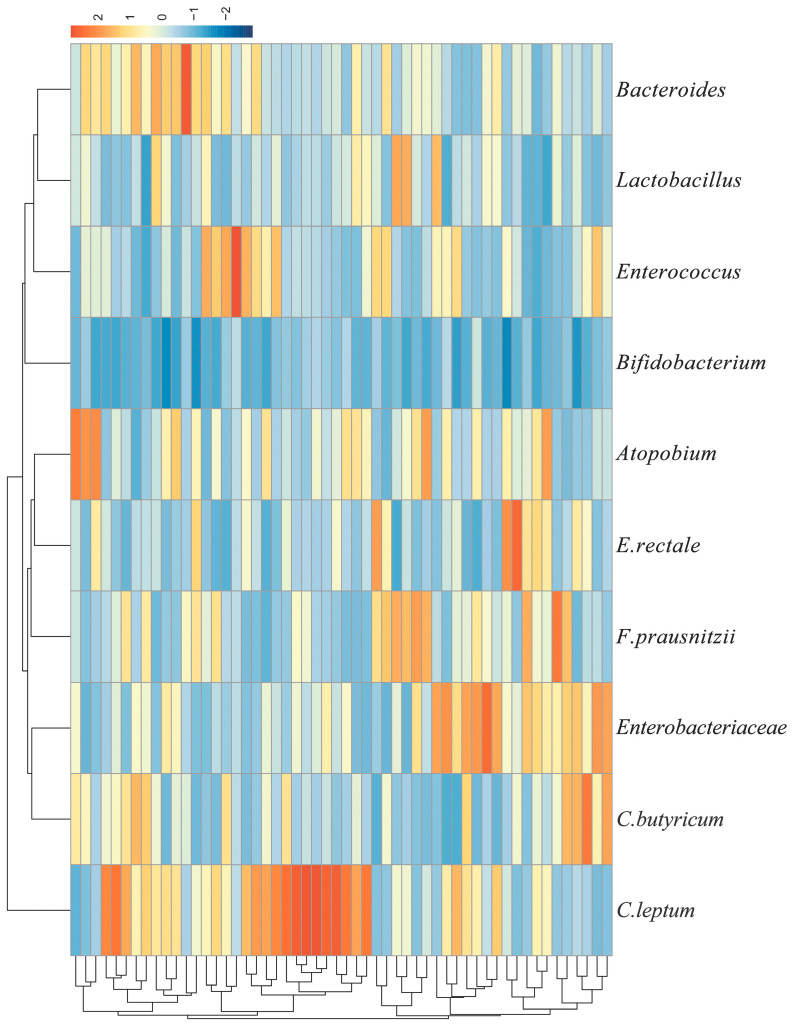
Gut microbiota abundance heatmap.

We used a heat map to visualize and explore the distribution of gut microbial abundance across participants (Figure 1). This graph was plotted based on Z-score normalized data, with the horizontal axis representing different genera of gut microbiota (e.g., *Bacteroides*, *Lactobacillus*, *Enterococcus*, *Bifidobacterium*, etc.), and the vertical axis representing different samples, with the transition of color from red to blue representing the relative abundance from high to low. The hierarchical clustering analysis showed that there were certain patterns of clustering in the microbial composition of different samples, revealing that certain samples may have similar gut microbial characteristics. In addition, clustering patterns were observed among certain microbial species. Specifically, *Bacteroides* clustered closely with *Lactobacillus*, *Atopobium* with *E. rectale*, and *Enterobacteriaceae* with *C. butyricum*. This suggests that these pairs of microbes exhibit similar abundance patterns across the samples, indicating potential associations in their ecological relationships or co-occurrence within the gut microbiota.

(2) Correlation of specific gut microbes with host anxiety, stress, and sleep quality scores

**Figure 2:**
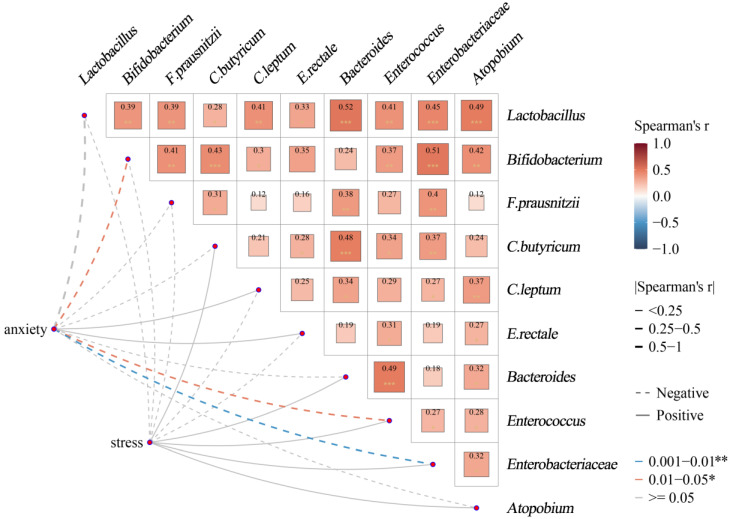
Association between gut microbiota and anxiety and stress: Spearman’s correlation network and heatmap. * *p* < 0.05, ** *p* < 0.01, *** *p* ≥ 0.05.

To explore the relationship between gut microbes and host anxiety, stress, and sleep quality in more depth, we performed a Spearman correlation analysis and visualized it using a correlation network heat map (Figure 2). The horizontal and vertical axes of the correlation network heat map represent the specific gut microbes studied. The squares in the heat map represent the Spearman correlation coefficients between the relative abundance of two gut microbiota. The squares have two elements, color and size: the color transitions from blue to red, representing the transition of the Spearman’s r from a negative to positive correlation, with red being a positive correlation and blue being a negative correlation, with lighter colors representing smaller absolute values of Spearman’s r and smaller squares; darker colors show that the larger the absolute value of Spearman’s r, the larger the correlation, and the larger the square. The lines connecting the graphs reveal the results of the Spearman’s correlation analysis of host anxiety and stress levels with ten gut microbes. The dashed line represents a negative correlation, and the solid line represents a positive correlation. The thickness of the line represents the size of the absolute value of Spearman’s r. The thicker the line, the larger the absolute value of Spearman’s r. The colors of the lines represent differences in significance, with gray representing that the correlation is not significant (*p* ≥ 0.05) and orange and blue representing that the correlation is significant (*p* < 0.05).

The results of the analysis showed that the relative abundance of *Bifidobacterium*, *Enterobacteriaceae*, and *Enterococcus* among the gut microbiota tested was negatively correlated with the level of host anxiety (Spearman’s r = −0.41, *p* < 0.05; Spearman’s r = −0.39, *p* < 0.05; Spearman’s r = −0.45, *p* < 0.01); gut microbiota may be associated with the onset of anxiety, whereas for the other microbiota, we did not observe a correlation of significance (*p* > 0.05). Further, we did not observe any gut microbiota that were significantly associated with stress levels (*p* > 0.05). This pattern of microbial co-occurrence suggests that gut microbiota may influence host’s state of anxiety, potentially revealing an important role for the microbiota–gut–brain axis in anxiety and stress regulation. In addition, we observed a correlation between different microbiotas. For example, *Lactobacillus* and *Bacteroides* abundances show a high positive correlation (Spearman’s r = 0.52, *p* < 0.01) and *Bifidobacterium* and *Enterobacteriaceae* show a high positive correlation (Spearman’s r = 0.51, *p* < 0.01). This positive correlation may reflect that the presence of one microbe promotes an increase in the abundance of another. This demonstrates that there are certain ecological interactions between different gut microbes, which can help us better explore their impact on host health.

(3) Correlation of specific gut microbes with host sleep quality and sleepiness scores

**Figure 3:**
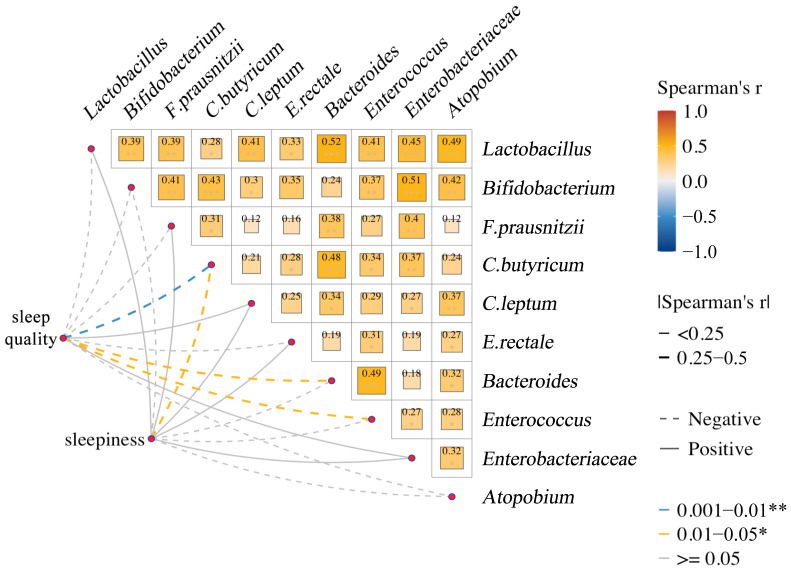
Association between gut microbiota and sleep quality and sleepiness: Spearman’s correlation network and heatmap. * *p* < 0.05, ** *p* < 0.01, *** *p* ≥ 0.05.

We also explored the relationship between gut microbiota and host sleep quality and sleepiness, and we similarly performed Spearman’s correlation analyses and visualized them using a correlation network heat map (Figure 3). The color of the squares in the heatmap transitioned from blue to orange, representing the transition of Spearman’s r from a negative to positive correlation, with red being a positive correlation and blue a negative correlation, with lighter colors representing smaller absolute values of Spearman’s r and darker colors representing larger absolute values of Spearman’s r. The lighter the color, the smaller the absolute value of Spearman’s r; the darker the color, the larger the absolute value of Spearman’s r. The rest is consistent with the explanation of Figure 2 and will not be described here. We found that *C. butyricum*, *Bacteroides*, and *Enterococcus* were negatively correlated with higher sleep quality (Spearman’s r = −0.61, *p* < 0.01; Spearman’s r = −0.41, *p* < 0.05; Spearman’s r = −0.42, *p* < 0.05), whereas for the other microbes, we did not observe a significant correlation with sleep quality (*p* > 0.05). We also observed a negative correlation between *C. butyricum* and higher levels of sleepiness (Spearman’s r = −0.44, *p* < 0.05), while for the other microbes, we did not observe a correlation of significance with sleepiness (*p* > 0.05). The results suggest that the high abundance of microbiota may be associated with decreased host sleep quality.

(4) Differences in gut microbiome composition between anxiety, stress, sleep quality, and sleepiness subgroups

**Figure 4:**
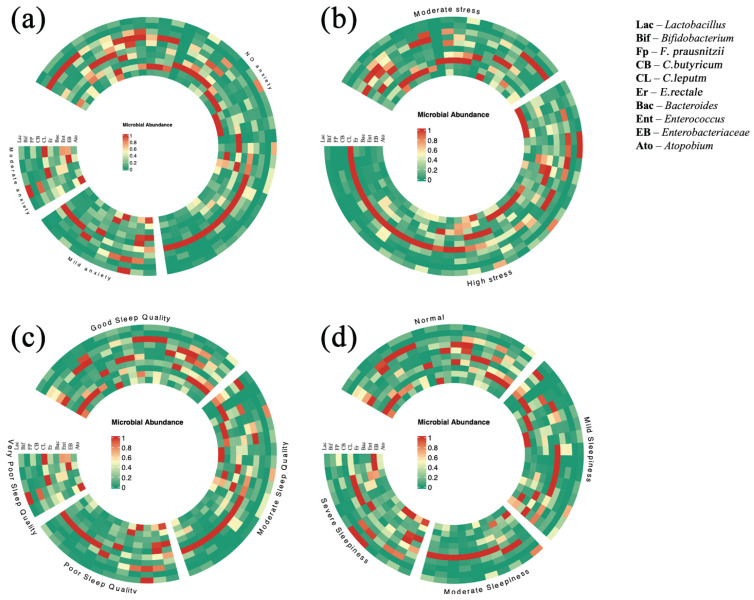
Circular heatmap of gut microbiota composition across different (**a**) anxiety groups, (**b**) stress groups, (**c**) sleep quality groups, and (**d**) sleepiness groups.

To visually demonstrate the distribution of gut microbes in relation to different levels of anxiety, stress, sleep quality, and sleepiness in hosts, we separated the samples into three groups based on the anxiety scale score (<50 no anxiety, 50–59 mild anxiety, 60–69 moderate anxiety); two stress groups based on the PSS score (14–18 moderate stress, 19–25 high Stress); four sleep quality groups based on the PSQI score (0–5 good sleep quality, 6–10 moderate sleep quality, 11–15 poor sleep quality, 16–21 very poor sleep quality); and four groups based on the ESS score (0–5 normal, 6–10 mild sleepiness, 11–15 moderate sleepiness, 16–24 severe sleepiness). Subsequently, we plotted grouped heatmaps for each of the variables for visualization (Figure 4). Each circle was grouped by anxiety level, stress level, sleep quality level, and sleepiness level; the different groups were arranged in separate sectors, each vertical row represented each participant sample, and each inner ring represented a specific gut microbe, with the ten gut microbes studied corresponding to the ten inner rings. The color transition from dark red to dark green reflects the relative abundance of gut microbiota, with darker red indicating higher abundance and darker green indicating lower abundance.

In the anxiety subgroup ring heat map (a), we observed that the anxiety-free group showed a higher abundance of *C. leptum*, *F. prausnitzii*, *Enterobacteriaceae*, and *Enterococcus* than the remaining two groups. To further explore the group differences, for variables that meet the assumptions of normality and homogeneity of variance, we applied a one-way ANOVA. For variables that do not meet these assumptions, we employed the Kruskal–Wallis H test. The results indicated a significant statistical difference in *Enterobacteriaceae* between the different anxiety groups (F = 3.21, *p* < 0.05), while no significant differences were found for the other microbes (*p* > 0.05). To identify which groups showed significant differences in *Enterobacteriaceae*, we conducted the LSD test. The results revealed a significant statistical difference in *Enterobacteriaceae* between the no anxiety and moderate anxiety groups, with a higher abundance observed in the no anxiety group (0.76, 95% CI: 0.15–1.37, *p* = 0.015). There was a significant statistical difference in *Enterobacteriaceae* between mild anxiety and moderate anxiety groups, with a higher abundance observed in the mild anxiety group (0.71, 95% CI: 0.15–1.41, *p* = 0.045). No significant statistical differences were found between the no anxiety and mild anxiety groups (*p* > 0.05). The no anxiety group had a more diverse microbial distribution than the moderate anxiety group. These results demonstrate that gut microbial abundance is also associated with host anxiety levels and that variations in the diversity of gut microbiota may accompany shifts in host anxiety levels.

In the stress subgroup circular heatmap (b), we observed that the moderate stress group had higher abundances of *F. prausnitzii*, *C. butyricum*, *Bacteroides*, and *Enterococcus* compared to the high stress group. To further explore the differences between the two groups, we performed independent samples *t*-tests on variables that followed a normal distribution and Mann–Whitney U tests on variables that did not follow a normal distribution. The results showed significant statistical differences in *Bacteroides*, with the high stress group exhibiting a higher abundance (−0.29, t = −2.02, 95% CI: −0.58–(−0.03), *p* < 0.05). No significant differences were observed for the other microbes (*p* > 0.05). In contrast, some participants in the high stress group exhibited notably lower diversity in their gut microbiota.

In the sleep quality subgroup circular heatmap (c), the group with good sleep quality showed higher abundances of *F. prausnitzii*, *C. butyricum*, and *Enterobacteriaceae* compared to the other groups with poorer sleep quality. The results showed significant statistical differences in *C. butyricum* (F = 4.05, *p* = 0.012), while no significant differences were observed for the other microbes (*p* > 0.05). To determine where the significant differences in *C. butyricum* abundance occurred between groups, we performed the LSD test. The results revealed significant statistical differences in *C. butyricum* abundance between the good sleep quality group and the poor sleep quality group, with the good sleep quality group exhibiting higher abundance (0.70, 95% CI: 0.24–1.16, *p* = 0.003). Additionally, significant statistical differences were found between the moderate sleep quality group and the poor sleep quality group, with the moderate sleep quality group having higher abundance (0.65, 95% CI: 0.19–1.11, *p* = 0.006). No significant statistical differences were observed between the other group comparisons (*p* > 0.05). The microbiota distribution in the good sleep quality group was more diverse compared to the other three groups, while in some samples from the moderate and poor sleep quality groups, the abundance of gut microbiota was notably lower.

In the sleepiness circular heatmap (d), the results showed significant statistical differences in *C. butyricum* (F = 3.72, *p* = 0.017), while no significant differences were observed for the other microbes (*p* > 0.05). To determine where the significant differences in *C. butyricum* abundance occurred between groups, we performed LSD tests. The results revealed significant statistical differences in *C. butyricum* abundance between the normal sleepiness group and moderate sleepiness group, with the normal sleepiness group exhibiting a higher abundance (0.61, 95% CI: 0.11–1.09, *p* = 0.018). There was a significant statistical difference in *C. butyricum* between the mild sleepiness group and severe sleepiness group, with the mild sleepiness group exhibiting a higher abundance (0.51, 95% CI: 0.03–0.99, *p* = 0.036). No significant statistical differences were observed between the other group comparisons (*p* > 0.05).

### 3.4. Gut Microbiota Composition Across Vitamin B1 and Vitamin B2 Groups

To explore the association of vitamin B1 and B2 levels with specific gut microorganisms, we used a grouped bubble chart to visualize the distribution of gut microbial abundance in different vitamin B1 and vitamin B2 groups (normal, insufficient, lacking). In the bubble chart, the horizontal axis has ten specific gut microorganisms, and the vertical axis represents the sample number. Each bubble represents the relative abundance of a gut microbe in a specific vitamin B1 or vitamin B2 level group; the size of the bubble reflects the level of abundance, with larger bubbles reflecting a greater abundance and smaller bubbles reflecting less abundance; the color of the bubbles indicates the grouping of different vitamin B1 or vitamin B2 levels.

**Figure 5:**
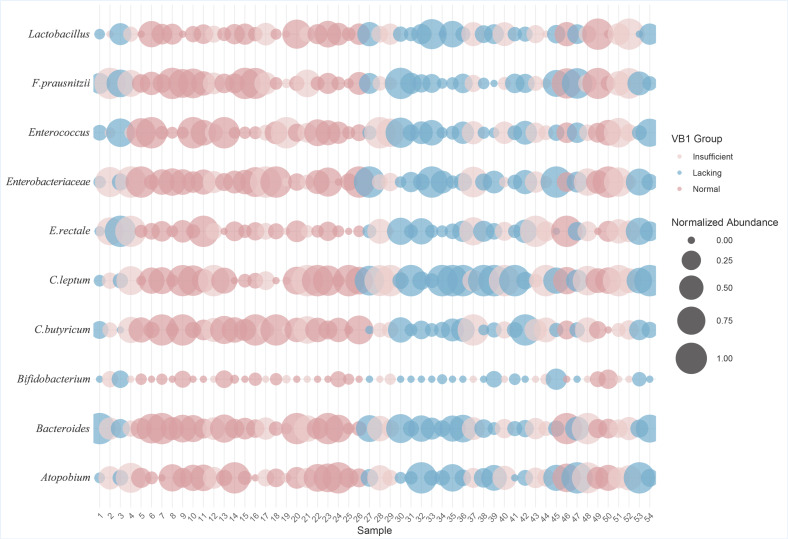
Bubble chart of gut microbiota abundance across different vitamin B1 levels.

Vitamin B1 grouping bubble plots show (Figure 5) that the groups had more individuals with normal and deficient levels of vitamin B1 and fewer individuals with deficiencies. The VB1 normal group presented a higher abundance of *Enterobacteriaceae*, *Bacteroides*, *C. butyricum*, *F. prausnitzii*, and *Enterococcus* than the insufficient and lacking groups. What caught our attention was that Bifidobacterium abundance was significantly lower than the remaining nine microbes. To further explore the differences between groups, we performed the Kruskal–Wallis H test. The results showed that there were no significant statistical differences in the abundances of ten gut microbiota species among the different vitamin B1 groups (*p* > 0.05).

**Figure 6:**
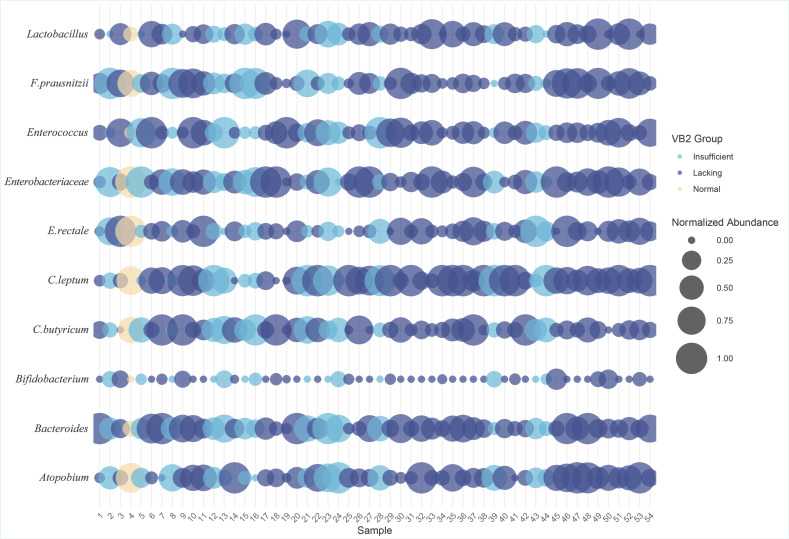
Bubble chart of gut microbiota abundance across different vitamin B2 levels.

The vitamin B2 grouping bubble plot showed (Figure 6) that participants had more individuals with deficient and insufficient levels of vitamin B2 and fewer normal individuals. We observed that the group lacking in vitamin B2 presented a higher abundance of *F. prausnitzii*, *Bacteroides*, *Atopobium*, *Enterobacteriaceae*, and *C. leptum* than the insufficient and normal groups. To further explore the differences between groups, we performed the one-way ANOVA for variables that meet the assumptions of the ANOVA. For variables that do not meet these assumptions, we employed the Kruskal–Wallis H test. The results show that there were no significant statistical differences in the abundances of ten gut microbiota species among the different vitamin B2 groups (*p* > 0.05).

### 3.5. Analysis of Mediating Effects of the Vitamin B1/B2 → Gut Microbes → Anxiety/Stress/Sleep Quality Pathway: Using a Path Analysis Framework

In the previous section, we observed multiple associations between individual vitamin B1 and B2 levels, gut microbiota, and host mental health (anxiety, stress, sleep quality, and sleepiness), and the link between the three variables piqued our interest. Based on the microbiota–gut–brain axis, we wanted to explore whether vitamins (VB1/VB2) could influence host mental health (anxiety, stress, sleep quality, and drowsiness) via gut microbes. A mediation effect is the effect of an independent variable (X) on a dependent variable (Y) through a mediating variable (M), denoted as pathway X → M → Y. Therefore, we conducted mediation effect analyses for two pathways: (1) vitamin B1 → gut Microbiota → mental health (anxiety, stress, sleep quality, and sleepiness); and (2) vitamin B2 → gut microbiota → mental health (anxiety, stress, sleep quality, and sleepiness).

We conducted a mediation effect analysis based on a path analysis framework and implemented it using SEM syntax. According to research by Wen et al., prior to conducting the mediation analysis, preliminary tests were performed to confirm significant associations among the independent variable, the potential mediator, and the outcome variable [39]. This step ensures that the conditions for mediation are met. The mediation models were implemented within a path analysis framework using SEM syntax (lavaan package in R, version 0.6–19). The basic condition for conducting mediated effects was that both correlation and regression analyses between paths X → M and M → Y were significant (*p* < 0.05). In pathway 1, we performed correlation and regression analyses between vitamin B1 and gut microbes and gut microbiota and mental health scores (anxiety, stress, sleep quality, and sleepiness). The results of the correlation analysis and regression were as follows. (1) X → M: In the correlation analysis between vitamin B1 and ten gut microbiota, vitamin B1 was significantly correlated with *C. butyricum* (Spearman’s = 0.28, *p* < 0.05) and insignificantly correlated with other microbes (*p* > 0.05). However, in the linear regression analysis, none of them regressed significantly (*p* > 0.05). (2) M → Y: In the correlation analysis between gut microbes and mental health scores (anxiety, stress, quality of sleep, and sleepiness), anxiety was correlated significantly with *Bifidobacterium* and *Enterobacteriaceae* (Spearman’s = −0.41, *p* < 0.05; Spearman’s = −0.36, *p* < 0.05); sleep quality correlated significantly with *C. butyricum*, *Bacteroides*, and *Enterococcus* (Spearman’s = −0.61, *p* < 0.05; Spearman’s = −0.41, *p* < 0.05; Spearman’s = −0.42, *p* < 0.05); and sleepiness scores correlated significantly with *C. butyricum* (Spearman’s = −0.44, *p* < 0.05). In regression analyses, only the regression of sleep quality with *Bacteroides* was significant (*p* < 0.05). In pathway 2, we performed correlation and regression analyses between vitamin B2 and gut microbiota and mental health scores (anxiety, stress, sleep quality, and sleepiness). The results of the correlation analysis and regression were as follows. (1) X → M: Vitamin B2 correlated significantly with *Bacteroides* (*p* < 0.05) and insignificantly with other gut microbes (*p* > 0.05). In the linear regression analysis, the regression relationship between vitamin B2 and *Bacteroides* was significant (*p* < 0.05). (2) M → Y: The variables were the same as in pathway 1, and the results are not described further. Overall, path 1 did not fulfill the basic conditions for mediation effects and was not included in the mediation effects analysis. However, the correlation and regression between vitamin B2 and *Bacteroides* were significant (*p* < 0.05), and the correlation and regression analyses between *Bacteroides* and sleep quality were significant (*p* < 0.05), which fulfilled the conditions for constructing the mediation model of VB2 → *Bacteroides* → sleep quality.

**Figure 7:**
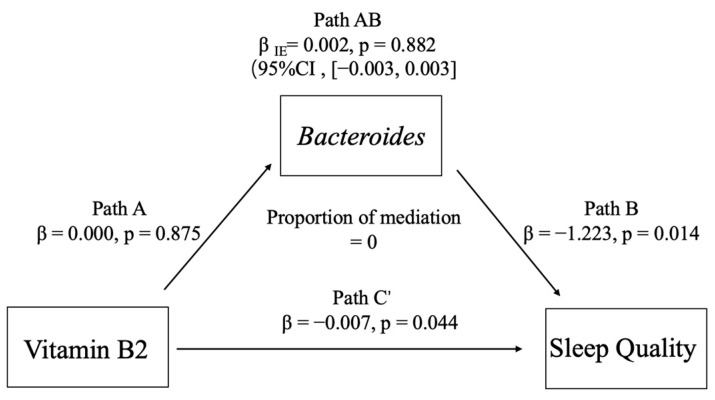
Mediation analysis of the effect of vitamin B2 on sleep quality via Bacteroides.

The results of the mediated effects analysis (Figure 7) showed that (1) the direct effect of pathway A (VB2 → *Bacteroides*) was not significant (β = 0.000, *p* = 0.875), suggesting that there was no direct effect of VB2 on the abundance of *Bacteroides*; (2) the direct effect of pathway B (*Bacteroides* → sleep quality) was significant (the direct effect of pathway B (*Bacteroides* → sleep quality) was significant (β = −1.223, *p* = 0.014)), suggesting that *Bacteroides* may play an important role in regulating the quality of sleep, and (3) the direct effect of pathway C’ (VB2 → sleep quality) was also significant (β = −0.007, *p* = 0.044), suggesting that VB2 has a direct effect on the quality of sleep, which may not be necessarily mediated by the mediator variable of *Bacteroides.* However, *Bacteroides* may not necessarily act through the mediating variable. The indirect effect analysis (path A × B) was not significant (β = 0.002, *p* = 0.882) and the Bootstrap confidence interval [−0.003, 0.003] contained 0, further suggesting that *Bacteroides* is not a key mediator of VB2 on sleep quality.

## 4. Discussion

### 4.1. Summary of Key Findings

Based on this study, we examined the interactions between vitamin B1 and B2 levels, specific gut microbiota, and host psychological states such as anxiety, stress, and sleep within the framework of the microbiota–gut–brain axis. Results showed a positive correlation between vitamin B1 level with stress scores and a negative correlation with sleep quality and sleepiness scores. Vitamin B2 levels showed a negative correlation with sleepiness scores. These findings demonstrated that both vitamins may play a role in modulating stress and sleep in the host. We also found a positive correlation between anxiety and stress scores, and a positive correlation between sleep quality and sleepiness scores. These results indicate potential interactions between host anxiety and stress status, and between sleep and sleepiness. In the analysis of gut microbiota and psychological states (anxiety, stress, sleep), we found that the microbiota–gut–brain axis may play a key role in the regulation of host psychological states, especially microbes such as *Bifidobacterium*, *Enterobacteriaceae*, and *Enterococcus*, which showed a negative correlation with host anxiety scores; *C. Butyricum*, *Bacteroides*, and *Enterococcus* showed a negative correlation with the host sleep quality index, and *C. Butyricum* demonstrated a negative correlation with host sleepiness scores. Additionally, *Lactobacillus* and *Bacteroides* abundances show a positive correlation and *Bifidobacterium* and *Enterobacteriaceae* show a positive correlation. By grouping psychological states, we further explored the differences in gut microbial abundance in individuals with different psychological state levels. A higher abundance of *Enterobacteriaceae* was found in the no anxiety group compared to the moderate anxiety group; a higher abundance of *Enterobacteriaceae* was found in the mild anxiety group compared to the moderate anxiety group; the high stress group showed a higher abundance of *Bacteroides* than the moderate stress group; a higher abundance of *C. Butyricum* was found in the good sleep quality group compared to the poor sleep quality group. A higher abundance of *C. Butyricum* was found in the moderate sleep quality group compared to the poor sleep quality group. The normal sleepiness group demonstrated a higher abundance of *C. Butyricum* than the moderate sleepiness group. The mild sleepiness group demonstrated a higher abundance of *C. Butyricum* than the severe sleepiness group. In the analysis of gut microbiota and vitamin levels, there were no significant statistical differences in the abundances of ten gut microbiota species among the different vitamin B1 and vitamin B2 groups. At the same time, the results showed that hosts with different psychological conditions, sleep, and vitamin B1 and B2 levels also indicated different abundances of gut microbes. In the mediation analysis, both *Bacteroides* and vitamin B2 showed significant direct effects on sleep quality. However, the mediating effect of *Bacteroides* in the association between vitamin B2 and sleep quality was not statistically significant.

### 4.2. Examining the Findings in the Context of the Existing Literature

Thiamine (vitamin B1) is essential for many physiological functions, including glucose metabolism, the maintenance of nerve membrane function, and the synthesis of myelin and several types of neurotransmitters (such as acetylcholine, serotonin, and amino acids) [40]. The most important function of thiamine is believed to be its significant role in cellular energy metabolism [41]. These neurological functions play an important role in sleep. A Korean national health and nutrition examination survey also showed that a thiamine deficiency was associated with poor sleep quality [42]. This supports the findings of this study, according to which vitamin B1 levels are associated with improved sleep quality and improved sleepiness. Previous reports have suggested that a significant reduction in serotonin level increases the responsiveness to stress [43]. An animal study showed that mice exposed to thiamine-deficient levels had significantly reduced serotonin levels [44]. In contrast, this finding is inconsistent with the positive correlation observed in this study between vitamin B1 levels and stress status. Similarly, a cross-sectional study reporting on the relationship between vitamin B1 intake and stress levels in 7387 Iranian adults found no significant association between vitamin B1 intake and stress levels [18]. This will require more in-depth research in the future to explore the potential relationship between stress and thiamine.

Vitamin B2 is a precursor for the synthesis of two coenzymes, FMN (Flavin Mononucleotide) and FAD (Flavin Adenine Dinucleotide), which play a key role in cellular energy metabolism and ATP production [45]. With the participation of riboflavin cofactor, glutathione reductase reduces oxidized glutathione (GSSG) to reduced glutathione (GSH), which can eliminate free radicals, alleviate oxidative stress, and protect cells from damage [46]. The antioxidant capacity of riboflavin is crucial for sleep quality [47]. An animal study reported that the hypermetabolism and immunopathology in sleep-deprived animals are expected to produce excessive metabolic and oxidative burdens [48]. Research has reported that the adequate dietary intake of vitamin B2 is associated with better sleep [49]. This supports the findings of this study, according to which vitamin B2 levels are associated with an improved sleepiness status. There is a strong bidirectional relationship between anxiety and stress behaviors and their neurological basis [50]. The most common cause of excessive sleepiness may be interference with sleep quality, sleep quantity, or other factors. The most common cause of this symptom is insufficient sleep [51]. This supports the findings of this study, namely that higher anxiety is associated with higher stress, while poorer sleep quality is associated with more severe sleepiness.

Serotonin (5-HT) and gamma-aminobutyric acid (GABA) are two neurotransmitters that the microbiota–gut–brain axis can use to influence host mental health. In vitro experiments have shown that certain microbial strains can even synthesize serotonin from tryptophan [e.g., *Lactobacillus plantarum* (FI8595)], which may determine 5-HT synthesis. Although 5-HT is believed to be crucial in the control of anxiety, stress, and depression, current neuroscience indicates that its relationship to anxiety depends on brain regions, receptor types, and even personal characteristics [52,53]. This supports the findings of this study, according to which the composition and abundance of gut microbes are associated with host anxiety levels, which may be relevant to the interaction between gut microbes and 5-HT levels. Certain *Lactobacillus* and *Bifidobacterium* strains have the capacity to generate GABA, according to an in vitro experiment that cultured strains of human gut origin [54]. Changes in central GABA receptor expression are linked to the pathophysiology of anxiety and depression, as evidenced by some animal studies that show *Lactobacillus* strains can modulate central GABA receptor expression via the vagus nerve in mice and reduce stress-induced corticosterone as well as anxiety- and depression-related behaviors [29]. In our study, we found that a higher *bifidobacteria* abundance was associated with lower anxiety levels in hosts, consistent with previous research findings. This may be associated with the ability of *bifidobacteria* to produce GABA, thereby affecting anxiety levels.

The microbiota–gut–brain axis can affect the host’s mental health and quality of sleep through short-chain fatty acids. Acetate, propionate, and butyrate make up the majority of short-chain fatty acids [55]. Numerous studies have demonstrated how important short-chain fatty acids are for gut–brain axis transmission. Short-chain fatty acids (SCFAs) support gut health by fueling intestinal epithelial cells, enhancing barrier function, and regulating metabolism [56]. They also influence the brain by crossing the blood–brain barrier, modulating microglial function, participating in inflammation, and affecting neurotransmitter and potassium levels [57]. Animal studies have shown that colonization with butyrate or butyrate-producing strains enhances blood–brain barrier integrity [58], and supplementing germ-free mice with SCFAs can restore microglial gene expression, number, and morphology [59]. Some gut microbiota genera have been shown to be capable of producing short-chain fatty acids. Certain gut microbiota genera, such as *Clostridium spp.* and *C. butyricum*, can produce butyrate in vitro [60], while some other taxa produce succinate, a precursor of propionate [61]. Our study results indicate that the abundance of *C. butyricum* in the good sleep quality group was higher than that in the group with poor sleep quality. Additionally, the abundance of *C. butyricum* in the moderate sleep quality group was also higher than that in the poor sleep quality group. Furthermore, participants with normal sleepiness had a higher *C. butyricum* abundance than those with moderate sleepiness. The mild sleepiness group had a higher *C. butyricum* abundance than the severe sleepiness group. This may be related to their ability to produce SCFAs, which is consistent with our study findings.

Sleep quality and host mental health are intimately linked to gut homeostasis. It is acknowledged that gut microbiota and their hosts have a complex and advantageous symbiotic connection [62]. A macro-genome sequencing study of human gut and fecal samples has shown that 98% of the gut microbiota is made up of the *phylums Thick-walled Bacteria*, *Bacteroidetes*, *Ascomycetes*, and *Actinobacteria* [63]. Because they consume nutrients and produce molecules that inhibit their growth to defend against pathogenic bacteria [64], modulate the immune system [65], and maintain barrier functions [66], these resident gut microbes are essential to gut homeostasis and host health. In addition, gut dysbiosis may impact host anxiety and stress via the gut–brain axis and is frequently linked to host chronic gut immunological diseases and other gastrointestinal disorders. Numerous gut and external factors also affect gut homeostasis, including host exposure to short-term stress, which can also affect microbial community profiles by changing the relative proportions of the major microbiota phyla [67], and individual aging, which causes the gut microbiota to become unstable and less diverse, along with decreased immune function [68]. Based on certain research, some commensal *clostridia* are essential for maintaining and creating gut homeostasis [69]. This study found that the gut microbial diversity was higher in the no anxiety group than in the mild and moderate anxiety groups, and it was higher in the good sleep quality and normal sleepiness groups than in the sleepiness and poorer sleep quality groups. This is consistent with previous research findings. This demonstrates that the diversity and balance of gut microbiota may interact and be linked to the psychological state of the host.

In addition, we also observed ecological interactions between different gut microbes. Bacteroides are well known for inhibiting anaerobic bacterial infections, but when they remain in the gut, they maintain complex and typically beneficial relationships with the host [70]. An animal study demonstrated that the oral administration of *Lactobacillus (L. johnsonii)* and *Bacteroides (B. thetaiotaomicron)* in DSS-induced colitis mice suppressed the overgrowth of *Escherichia coli*, *Enterococcus faecalis*, and *Candida albicans*, accompanied by significant reductions in inflammatory markers [71]. In the current study, *Lactobacillus* and *Bacteroides* abundance showed a significant correlation, suggesting potential ecological interactions. This is consistent with the results mentioned earlier. Additionally, *Enterobacteriaceae* are typically associated with inflammatory responses [72], while *Bifidobacterium* is considered beneficial to human host health [73]. This is inconsistent with the results of this study, which showed a positive correlation between *Enterobacteriaceae* and *Bifidobacterium*. In the complex intestinal environment, there may be a symbiotic and metabolic interaction between *Bifidobacterium* and *Enterobacteriaceae*, rather than just a competitive relationship. Various studies have demonstrated that some members of the *bifidobacterial* community can co-operate to degrade large and complex polysaccharides into more simple sugars, which are in turn then available to other members of the gut microbiota [74]. This is worth exploring further in the future.

Our findings indicate that *Bacteroides* does not serve as a statistically significant mediator in the relationship between riboflavin levels and sleep quality. Although both riboflavin levels and *Bacteroides* showed significant direct associations with sleep quality, these effects do not appear to be mediated through *Bacteroides*. Previous studies have shown that all *Bacteroidetes* genomes possess the complete core functionality for riboflavin biosynthesis, meaning that they are capable of the de novo synthesis of riboflavin [24]. Compared with the *Actinobacteria* and *Bacillariophyceae* phyla, the ecological role of riboflavin synthesis in *Bacteroidetes* may be more stable and complete [24]. At the same time, riboflavin deficiency can also affect intestinal morphological changes [75]. However, these mechanistic insights do not align with our empirical results. It is important to note that the interplay between riboflavin and gut microbiota may be modulated by multiple factors, including host lifestyle, dietary patterns, and individual genetic variability [76]. These potential confounders may obscure detectable mediation effects. Therefore, our preliminary findings are insufficient to support the hypothesis that *Bacteroides* mediates the relationship between riboflavin and host sleep quality. Future studies with larger sample sizes and integrated multi-omics approaches are needed to further elucidate the complex interactions among gut microbiota, vitamin B metabolism, and psychological health.

### 4.3. Limitations and Future Directions

This study is cross-sectional in design, which limits its ability to establish causal relationships between gut microbiota composition, mental health status (such as anxiety, stress, and sleep quality), and vitamin B1/B2 levels. Additionally, due to the small sample size, the generalizability and representativeness of some statistical results might be limited. Future studies with larger samples, using longitudinal cohort studies or randomized controlled trials, could provide further insights into the evidence of potential causal associations. This study did not apply corrections for multiple comparisons, which may increase the risk of false-positive findings. Therefore, the results should be interpreted as exploratory and require further validation in future studies. Secondly, according to ethical consideration, this study was unable to measure the activity of erythrocyte thiamine pyrophosphate-dependent enzyme (ETKAC) or the concentrations of erythrocyte riboflavin and its derivatives. Additionally, key biomarkers closely associated with the gut microbiota, such as lipopolysaccharide (LPS), 5-HT, and SCFAs, were not assessed. Future studies should incorporate these molecular markers to provide a more comprehensive understanding of the potential mechanisms underlying the microbiota–gut–brain axis. Finally, this study was conducted on a relatively small human sample and quantified only 10 selected gut microbial taxa, without performing comprehensive microbiota profiling, such as 16S rRNA or metagenomic analysis. Future research employing a multi-omics approach could thoroughly examine the impact of the gut–brain axis on host anxiety, stress, and sleep quality.

Furthermore, although this research was conducted among a relatively common population of college students in China, the results may not be entirely applicable to other populations due to differences in nutritional structure, genetic background, and environmental factors. To improve the data’ generalizability and extrapolability, future research should include populations with varying dietary models, different ages, and health conditions. It should also be noted that this study may be influenced by potential confounding factors such as dietary, lifestyle, sleep, and physical activity levels. Psychological outcomes in this research were assessed using self-reported questionnaires, which may be subject to a self-report bias. These factors may affect the accuracy and objectivity of mental health assessments. Future research could also use microbiomics to investigate whether antioxidant vitamins (e.g., vitamin C and vitamin D) and other gut microbiota (like *Archaea intestinalis*, *Aspergillus phylum*, *Micrococcus wartyi*, etc.) have a greater influence on how vitamin levels impact stress, anxiety, and sleep quality. A key strength of this study lies in its use of a population-based sample to preliminarily explore the associations among gut microbiota, host anxiety, stress, sleep, and vitamin B1 and B2 levels. The findings provide preliminary human-based evidence to support future applications of microbiota-targeted therapies, such as probiotic supplementation or fecal microbiota transplantation (FMT), as well as precision nutrition strategies aimed at reducing anxiety, alleviating stress, and improving sleep quality.

## 5. Conclusions

This study highlights the association between vitamin B1 and B2 levels, specific gut microbes, and host mental health, including anxiety, stress status, sleep quality, and sleepiness. The results indicate that higher vitamin B1 levels are associated with better sleep quality and reduced sleepiness, while higher vitamin B2 levels are linked to better sleepiness outcomes. These beneficial vitamin B1 and B2 levels may interact with human stress and sleep through their antioxidant properties. Additionally, this study found significant correlations between specific gut microbes and host anxiety, sleep quality, and sleepiness. The abundance of specific gut microbiota also showed significant inter-correlations. Significant differences in the abundance of specific gut microbes were observed between different anxiety, stress, sleep, and sleepiness groups. However, no significant differences in the abundance of specific gut microbes were found between different vitamin B1 and B2 groups. No significant mediating effect was observed among vitamin levels, gut microbes, and psychological health. Overall, this study, grounded in the gut–microbiota–brain axis framework, provides a preliminary exploration of the potential associations between vitamin B1 and B2 status, specific gut microbes, and host anxiety, stress, sleep quality, and sleepiness. However, the cross-sectional nature of the study limits the ability to infer causality. Future longitudinal and interventional studies are needed to further clarify the causal relationships among vitamins and the gut–brain axis. These findings offer exploratory insights for future research and highlight the need for more in-depth studies to elucidate the complex interactions among micronutrients, the gut microbiota, and mental health. Such efforts may contribute to understanding the potential pathway of the development of precision nutrition and microbiota-based interventions for improving psychological outcomes.

## Figures and Tables

**Table 1:** Baseline characteristics of participants.

Characteristic	Overall (*N* = 76)
Sociodemographic	
N	76
Age, y	19.5 ± 1.2
Sex, n(%)	
Female	36(47.4%)
Male	40(52.6%)
Education level, n(%)	
First year	48(63.1%)
Third year	28(36.8%)
Lifestyle	
BMI, kg/m^2^	21.3 ± 3.1
Daily physical activity practician, n(%)	
Mild level	64(84.2%)
Middle level	11(14.5%)
Severe level	1(1.3%)
Smoking, no, n(%)	69(90.7%)
Drinking, no, n(%)	70(92.1%)
Nutritional status	
Vitamin B1 in urine, ug	269.2 ± 382.8
Vitamin B2 in urine, ug	321.3 ± 365.5
Psychological status	
Anxiety score (SAS)	42.5 ± 9.9
Stress score (PSS)	19.6 ± 4.9
Sleep quality index (PSQI)	9.2 ± 4.8
Sleepiness score (ESS)	10.2 ± 5.7

Abbreviations: BMI, body mass index; data are presented as mean ± standard deviation (SD) for continuous variables and as frequency (percentage) for categorical variables.

**Table 2:** Results of independent sample *t*-test for vitamin B2 levels, anxiety, stress, sleep quality, and sleepiness between male and female participants.

Variable	t-Value	df	*p*-Value	Mean Difference	95%CI
Vitamin B2	−0.36	74	0.723	−13.74	−90.77–63.29
SAS	−3.78	74	<0.05 *	−8.08	−12.36–−3.79
PSS	−3.33	74	<0.05 *	−3.52	−5.62–−1.43
PSQI	−0.42	74	0.674	−0.48	−2.72–1.76
ESS	−0.48	74	0.631	−0.63	−3.23–1.97

* *p* < 0.05, indicating statistical significance.

**Table 3:** Effect of vitamin B1 levels on anxiety, stress, sleep quality, and sleepiness: Kruskal–Wallis test results.

Variable	df	H	*p*-Value
SAS	2	0.451	0.978
PSS	2	8.569	0.075
PSQI	2	34.087	<0.05 *
ESS	2	37.667	<0.05 *

* *p* < 0.05, indicating statistical significance.

**Table 4:** Effect of vitamin B2 levels on anxiety, stress, sleep quality, and sleepiness: one-way ANOVA results.

Variable	Sum of Squares	Mean Squares	F	*p*-Value
SAS	64.749	21.583	0.215	0.886
PSS	85.038	28.346	1.190	0.320
PSQI	177.761	59.254	2.641	0.056
ESS	311.028	103.676	3.561	0.018 *

* *p* < 0.05, indicating statistical significance.

**Table 5:** Spearman correlation analysis between vitamin B1 and vitamin B2 levels and scores of anxiety, stress, sleep quality, and sleepiness.

Variable	Vitamin B1	Vitamin B2	SAS	PSS	PSQI	ESS
Vitamin B1	1.000					
Vitamin B2	0.282 *	1.000				
SAS	0.002	−0.001	1.000			
PSS	0.341 *	0.149	0.357 *	1.000		
PSQI	−0.592 *	−0.223	0.191	−0.104	1.000	
ESS	−0.654 *	−0.235 *	0.060	−0.203	0.847 *	1.000

* *p* < 0.05, indicating statistical significance.

## Data Availability

The data presented in this study are available from the corresponding author on reasonable request, due to restrictions related to participant privacy.

## Supplementary Materials

The following supporting information can be downloaded at: https://www.mdpi.com/article/10.3390/nu17111894/s1, Supplementary Material A. Process of extracting total DNA from fecal microorganisms; Supplementary Material B. Primers and parameters used in this study; Supplementary Material C. Formula for calculating the final concentration of the target bacteria.
