# Supplementary material for: Host–Gut Microbiota Interactions: Exploring the Potential Role of Vitamin B1 and B2 in the Microbiota–Gut–Brain Axis and Anxiety, Stress, and Sleep Quality
Source: Nutrients. 2025 May 31;17(11):1894. doi: 10.3390/nu17111894 (PMC12158063; doi:10.3390/nu17111894)
Supplement: Supplementary file 1 [file nutrients-17-01894-s001.zip › nutrients-3611864-supplementary.pdf]

Supplementary Materials

Supplementary Material A. Process of extracting total DNA from fecal microorganisms

Extract and purify DNA from fecal samples using the Aidlab Fecal Genomic DNA Rapid Extraction Kit. Place the fecal sample (200-220 mg) in a centrifuge tube and add 1.4 ml of ASL buffer, vortexing continuously for one minute until the sample is completely mixed. The resuspension is placed in a warm bath at 70 degrees Celsius for 5 minutes; the heating step can help to lyse bacteria and parasites and increase DNA yield. Then, after vortexing and shaking for 15 seconds, place at room temperature for 1 minute and centrifuge at maximum speed for 3 minutes to remove impurities. Carefully transfer 900ul of supernatant to a 1.5ml centrifuge tube, add 100ul of Impurity Scavenger AB, immediately vortex and shake for 1 minute until completely mixed, and after being left at room temperature for 1 minute, centrifuge at maximum speed for 3 minutes to remove impurities. All supernatant was transferred to a 1.5 ml centrifuge tube and centrifuged again at maximum speed for 3 minutes. Transfer 210ul of supernatant to a new 1.5ml centrifuge tube, add 20ul of Proteinase K solution, add 200ul of Binding Solution CB, vortex and shake for 15 seconds, mix well and then warm bath at 70 degrees Celsius for 10 minutes. Remove the adsorbent column for pretreatment, add 100ul equilibrium solution, and centrifuge at 13000rpm for 1 minute. The centrifuge tube after the warm bath was cooled sufficiently, 100ul of isopropanol was added and vortexed and mixed. Add the solution obtained in the previous step and possible precipitation to the adsorbent column AC, centrifuge at 13,000 rpm for 30 seconds, and pour off the waste solution in the collection tube. Add 500ul of Inhibitor Removal Solution IR, centrifuge and pour off the waste solution. Add 600ul of Rinse Solution WB, centrifuge and pour off the waste solution and repeat the procedure twice. Place the column AC into an empty collection tube and centrifuge for 2 minutes to remove as much rinse solution as possible to avoid ethanol residue. Remove the column AC, put it into a clean centrifuge tube, add 100ul of eluent EB, leave it at room temperature for 2 minutes, centrifuge it at 12000rpm for 1 minute and repeat the procedure. the DNA can be stored at -20°C for a long time.

Supplementary Material B. Primers and parameters used in this study

Table S1. Primers used in this study

Target group	Pre-primer	Post-primer
Lactobacillus	GAGGCAGCAGTAGGGAATCTTC	GGCCAGTTACTACCTCTATCCTTCTTC
Bifidobacterium	CTCCTGGAAACGGGTGG	GGTGTTCTTCCCGATATCTACA
F. prausnitzii	GGAGGAAGAAGGTCTTCGG	AATTCCGCCTACCTCTGCACT
C. butyricum	ATGCAAGTCGAGCGATG	TATGCGGTATTAATCTTCCTTT
C. leptum	GCACAAGCAGTGGAGT	CTTCCTCCGTTTTGTCAA
E. rectale	CGGTACCTGACTAAGAAGC	AGTTTCATTCTTGCGAACG
Bacteroides	GAGAGGAAGGTCCCCCAC	CGCTACTTGGCTGGTTCAG
Enterococcus	CGTGCGAACATGACCGATAT	CGAAACGGCCATTAACCAAC
Enterobacteriaceae	GGTAGAGCACTGTTTCGGCA	TGTCTCCCGTGATAACATTCTC
Atopobium	GGGTTGAGAGACCGACC	CGGAGCTTCTTCTGCAGG

Table S2. qPCR thermal cycling conditions

Step	Temperature	Time	Cycles
Initial Denaturation	94℃	2 min	1
Amplification	94℃	10 sec	40
	60℃	20sec	40
	72℃	30sec	40
Dissociation Analysis	60℃-95℃	0.5℃ increment per cycle	1

Supplementary Material C. Formula for calculating the final concentration of the target bacteria

$$C\ (\text{copies}\cdot\text{ng}^{-1}) = \frac{N\ (\text{copies})}{C_t\ (\text{ng}\cdot\mu\text{L}^{-1}) \times V\ (\mu\text{L})}$$

- *Concentration of target bacteria (C)*
- *number of target bacteria (N)*
- *concentration of total DNA of fecal microorganisms (C_t)*
- *volume of the template (V)*
